# How political choices shaped Covid connectivity: The Italian case study

**DOI:** 10.1371/journal.pone.0261041

**Published:** 2021-12-10

**Authors:** Enrico Amico, Iulia Martina Bulai

**Affiliations:** 1 Institute of Bioengineering/Center for Neuroprosthetics EPFL—Ecole Polytechnique, Institute of Bioengineering Fédérale de Lausanne, Lausanne, Switzerland; 2 Department of Radiology and Medical Informatics, University of Geneva, Geneva, Switzerland; 3 Department of Mathematics, Computer Science and Economics, University of Basilicata, Potenza, Italy; Carlos III University of Madrid, SPAIN

## Abstract

The importance of implementing new methodologies to study the ever-increasing amount of Covid-19 data is apparent. The aftermath analysis of these data could inform us on how specific political decisions influenced the dynamics of the pandemic outbreak. In this paper we use the Italian outbreak as a case study, to study six different Covid indicators collected in twenty Italian regions. We define a new object, the Covidome, to investigate the network of functional Covid interactions between regions. We analyzed the Italian Covidome over the course of 2020, and found that Covid connectivity between regions follows a sharp North-South community gradient. Furthermore, we explored the Covidome dynamics and individuated differences in regional Covid connectivity between the first and second waves of the pandemic. These differences can be associated to the two different lockdown strategies adopted for the first and the second wave from the Italian government. Finally, we explored to what extent Covid connectivity was associated with the Italian geographical network, and found that Central regions were more tied to the structural constraints than Northern or Southern regions in the spread of the virus. We hope that this approach will be useful in gaining new insights on how political choices shaped Covid dynamics across nations.

## Introduction

The Covid-19 pandemic has produced an impressive amount of epidemiological data, collected all over the world [1]. Each country collected their data following different protocols depending on its respective national health service, [2–4]. In Italy, with twenty administrative regions independent on Health, Covid-19 data were made available at regional and national level trough the Italian Department of Civil Protection, [5]. The Italian Covid-19 collected data consists of time series or Covid indicators, such as: the number of hospitalized individuals in intensive care units (ICU), hospitalized individuals with symptoms, individuals in home isolation, new positives, discharged healed and deceased individuals. All of them are available for all twenty italian regions [5]. In the aftermath of the pandemic, these data provide a benchmark to investigate the effects of two distinct political decisions that were taken during the first and second wave of the Sars-Cov-2 spread, one at the beginning and the other at the end of 2020, respectively [6]. Specifically, the first Italian lockdown was a quite severe nationwide lockdown, whereas the second one was region-wide, tailored on each specific regional health situation [7].

The first Covid-19 pandemic wave took most countries and their leaders by surprise. The immediate reaction resulted in more severe policy interventions such as: travel bans, self-isolation, quarantines and stay-at-home orders; public-gathering and event restrictions; school, restaurant, and non-essential business closures; up to complete lockdowns [8]. The adopted restrictions were different between countries, but also, at a smaller scale, between regions and/or provinces/states. This differentiation eventually led to different results depending on the political decisions made [9]. Apart from improving the current epidemiological models for the SARS-CoV-2 transmission, researchers also focused on proposing alternative solutions to severe lockdown choices, in view of a potiental second (and third) wave, [10–12] by reducing some restrictions. These “soft lockdown” measures were also adopted by the Italian government, during the second wave of the pandemic.

In this work we tap into the link between these policy decisions and the network dynamics of the Sars-Cov-2 spread in Italy. In order to do so, inspired by methodology commonly used in brain network analysis [13], we introduce and analyze the “Covid functional connectome”, i.e. the *Covidome*, which is closely related to the covariance matrix of a specific Covid indicator. For instance, two italian regions that share a similar trend in the number of hospitalized in ICU will have high values in its correspondent Covidome values, and viceversa. In essence, the Covidome provides a summary picture of the pairwise “Covid connectivity” between nodes (the regions) of the Italian network, during the pandemic.

We use this representation to explore the Covidome community structure, in order to learn more about the hidden interactions between italian regions during the spread of the Covid-19 pandemic [14–16]. We found a specific North-South separation in two distinct “Covid functional” community, across almost all Covid indicators. Furthermore, using sliding window analysis, we found that Covid connectivity changed consistently across Northern, Central and Southern Italy, with major differences spiked by the regionwide lockdown for the second wave, on 4th of November 2020, and the more severe first lockdown, on 10th of March 2020. Notably, the measures introduced short before the second differentiated lockdown, i.e. the obligation to wear masks in open and closed public spaces (on 13th of October 2020), the closure of major non essential activities (on 24th of October 2020), etc., have led to evident concrete results, [17, 18]. In fact, Covid connectivity started decreasing already before the effective date of the second lockdown for all the time series considered, differently from the first lockdown where the correlation values started decreasing after the effective date. Finally, we investigate whether Covidomes related to the structural network of Italy (i.e. its geography). We found that Covid connectivity relates strongly to the structure more to central areas of Italy than to the Northern and Southern regions.

We believe that the innovation of analyzing Covid-19 time series as a complex structure of networked systems might help in the interpretation of the key political decisions in the aftermath. We hope that this approach will be useful in analyzing epidemiological data in general, and that this study might open up new research avenues able to gain new insights on how political choices can shape pandemic outbreaks.

## Materials and methods

In this section we will first introduce the time series (i.e., Covid indicators) used for this study, and detail the Italian outbreak and political decisions made to prevent it. Secondly, we will introduce the Covid connectivity matrix (”Covidome”) and give an overview of the network approaches employed.

### Data and outbreak details

The data used in this paper was collected by the Italian Department of Civil Protection and is freely available on a Github directory [5]. We analyzed different time series starting from 24 February 2020 until 7 January 2021 (few days after vaccine campaign started). All the considered time series are available for each Italian region. We focused on 6 different Covid indicators, such as: 1) the number of hospitalized individuals in ICU; 2) number of hospitalized individuals with symptoms; 3) number of individuals in home isolation; 4) number of new positives; 5) number of discharged healed; and 6) number of deceased individuals.

On February 20, 2020 the first severe patient was tested positive for SARS-Cov-2 at hospital of Codogno, Italy. Since this first episode a rapidly increasing number of patients have been identified, especially in the Northern part of the country. Italy was one of the most affected European country and was the first to implement drastic measures in the attempt to contain the disease. Below we list the most relevant dates for Italy (see [7] for more details):

- Lockdown of the Northern regions on March 8, 2020, which was followed by complete lockdown of Italy within a few days (10 March), including travel restrictions and a ban on public gatherings, [19].
- On March 22, the Italian government closed all non-essential businesses and industries, and restricted movement of people unless was strictly necessary, [20].
- On March 31, the president of the Italian National Institute of Health announced that the pandemic had reached its peak in the country, which corresponded to the start of the outbreak plateau. The news was confirmed also by the head of the Civil Protection Department.
- On April 20, Italy saw the first fall in the number of active cases.
- Covid-19 cases started to decline in May 2020, thanks to the two-months lockdown. Freedom of movements was re-established on May 4 and other not essential activities re-opened later that month, [21].
- On October 13 the obligation to wear masks, in both open and closed spaces, returns, [22], and on October 14, cases of Covid-19 positives exceeded the peak of the March infections.
- On October 18 new restrictions were applied with the possibility of distance learning for both high schools and universities depending on the regional epidemiological situation, [23].
- On October 24, major non essential activities were closed and distance learning political decisions were reapplied, [24].
- On November 4, the Italian Prime Minister announced a new lockdown, dividing the country into three zones depending on the severity of the pandemic, corresponding to red, orange and yellow regions. Moreover, a national curfew from 10 PM to 5 AM was implemented, as well as compulsory weekend closing for shopping malls, and online education in high schools, [25].
- From December 21 to January 6 further movement restrictions were implemented in order to prevent an increase in cases during the Christmas holidays period, and to block movement between regions, [26].

### Introducing the Covidome

We here define a “Covid connectivity network” (or *Covidome*). This network consists of 20 nodes, which corresponds to the Italian regions. For each of the six different aforementioned Covid indicators, the edge between region pairs is defined by its Pearson’s correlation coefficient (referred in the figures as Covid connectivity). Specifically, for two Covid time series *X* and *Y*, and *n* time points, this coefficient is defined as:

$$\begin{array}{c} r_{X,Y}=\frac{\sum_{i=1}^{n}\left(x_{i}-\bar{x}\right)\left(y_{i}-\bar{y}\right)}{\sqrt{\sum_{i=1}^{n}{\left(x_{i}-\bar{x}\right)}^{2}\sum_{i=1}^{n}{\left(y_{i}-\bar{y}\right)}^{2}}}. \end{array}$$ (1)

**Fig 1:**
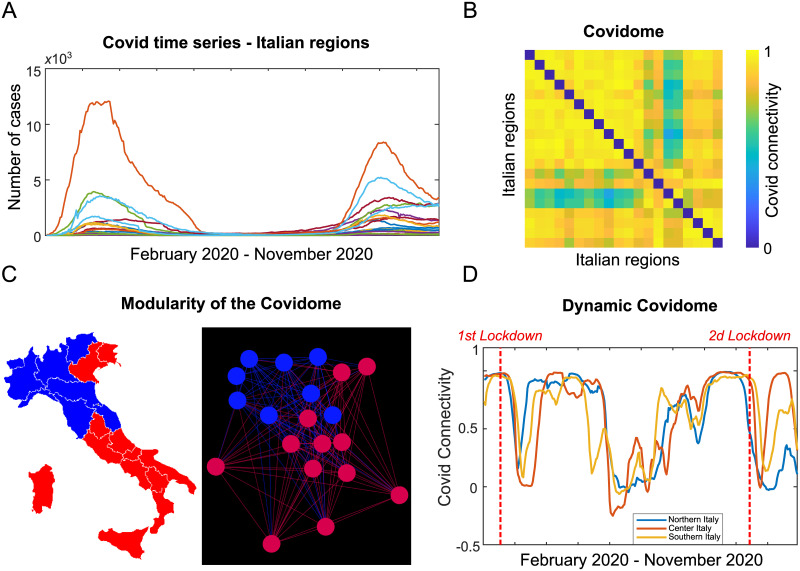
Workflow of the Covid connectivity analysis for hospitalized individuals with symptoms in Italy, during 2020. **A**. The time series of hospitalized individuals with symptoms for all the 20 Italian regions. **B**. The *Covidome* (the adjacency matrix of the network) obtained by computing the Pearson’s correlation coefficients associated to data reported in panel A. **C**. Communities of the Covidome for the considered time series, represented both on the Italian map (left panel) and on the graph (right panel), respectively. **D**. Average Covid connectivity obtained using sliding window correlation. The three different curves represent three different areas corresponding to Northern, Central and Southern Italy.

Once computed all the edges values we get six Covid-19 adjacency matrices, of dimension 20 × 20, that, for simplicity, will be referred to as Covidome throughout the text. In a nutshell, the Covidome represents a second-order statistic of the regional Covid trend reported by the time series evolution. In fact, it is closely related to the covariance matrix of the Covid indicators across Italian regions. Therefore, for each time series, high values in the Covidome will inform on two regions following the same trend in Covid dynamics, and viceversa. We will see in this paper how this information is tightly linked to the political decision made during 2020 to fight the pandemic spread. Please also see Fig 1B for an example of Covidome corresponding to the hospitalized individuals with symptoms time series (Fig 1A).

### Covidome modularity

We used the Newman and Girvan modularity score [14] to investigate the community structure of the Covidome. Given a network and a partition (modularity solution), the modularity score *Q* introduced in [14] is:

$$\begin{array}{c} Q_{score}=\frac{1}{2E}\sum_{ij}(A_{ij}-\gamma \frac{k_{i}k_{j}}{2E})\delta \left(m_{i},m_{j}\right), \end{array}$$ (2)

where *E* is the number of edges in the network, *A*_*ij*_ is the adjacency matrix of the network (in this case binary undirected obtained from the Covidome), *k*_*i*_ and *k*_*j*_ are the degree of nodes *i* and *j* respectively, *γ* the resolution parameter and *δ*(*m*_*i*_, *m*_*j*_) is the Kronecker delta between community *m*_*i*_ and *m*_*j*_. We used the Louvain algorithm [16] to obtain the optimal partition of the Covidome. To improve the robustness of the Louvain solution, we used the consensus clustering procedure introduced by Lancichinetti and Fortunato [15], by running Louvain 100 times and finding the optimal community solution obtained from the consensus matrix [13] over the 100 runs. Please see Fig 1C for an example of the community structure of the Covidome computed from the hospitalized individuals with symptoms time series. Finally, we also computed a Covid “allegiance matrix”, that is the probability that each region pair belonged to the same module across all Covid indicators. This matrix provides quantitative insights on whether two regions had similar Covid outbreaks (as reported by the six aforementioned indicators or time series) during the pandemic.

### Dynamic Covidome analysis

In order to better understand the link between the Covid-19 dynamics and the political decisions made, we performed sliding window analysis on the Covid indicators, inspired by techniques commonly used in Network Neuroscienc [13]. In a nutshell, we computed Covidome “snaposhots” (or dynamic Covidomes, where the Covidome was computed, as aforementioned, by using Pearson’s correlation coefficients as defined in (1)) at shorter time intervals within a sliding window of fixed length. We chose a three week window with a step (slide) of one day, to explore the Covidome dynamics at different time interval across 2020 (please see also the videos in Supplementary material). In order to investigate local differences in the dynamic Covidomes we first subdivided the Italian map in three main areas corresponding to Northern, Central and Southern Italy (see S1 Fig in S1 File for the geographical subdivision of the Italian regions), and then we evaluated the fluctuation of the mean value of the dynamic Covidomes (i.e., by considering the correlations with all the other regions (not necessarily in the same area), and then by computing the average correlation within area) across sliding windows (see Fig 1D).

## Results

The results reported in the next section are related to two complementary Covid indicators, such as the number of hospitalized individuals with symptoms and the number of new positives (with the exception of the Covid allegiance matrix, computed across all six time series, see Methods for details). For the results related to the remaining four Covid indicators (i.e., hospitalized individuals in intensive care units, individuals in home isolation, discharged healed and deceased individuals, respectively) please see the Supplementary material.

### Consensus modularity and allegiance matrix

**Fig 2:**
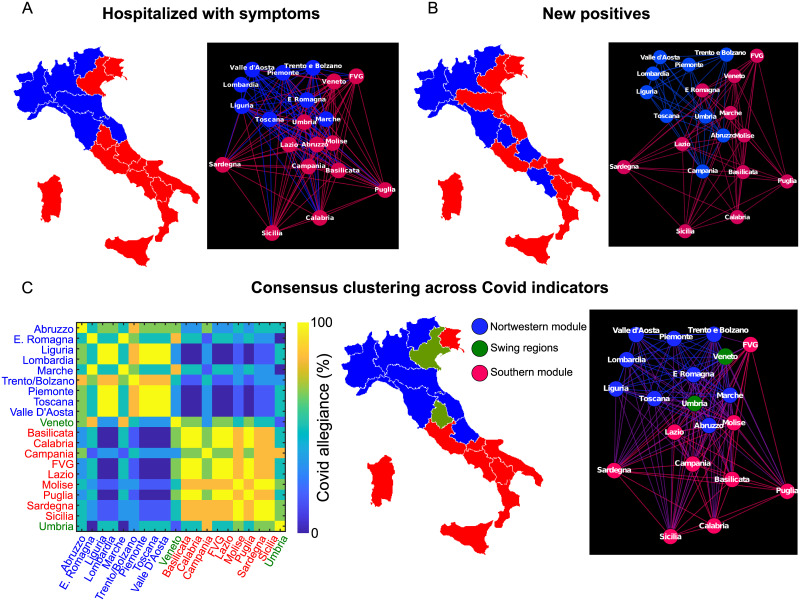
Community structure of the Italian Covidome. **A**. The Covidome partition, after consensus clustering, for the hospitalized with symptoms time series, represented on the map (left panel) and on the Covidome graph (right panel). **B**. The Covidome partition, after consensus clustering, for the new positives time series, on the map (left panel) and on the Covidome graph (right panel). **C**. The Covidome allegiance matrix (left panel) across the six different Covid indicators (i.e., number of hospitalized individuals in ICU, number of hospitalized individuals with symptoms, number of individuals in home isolation, new positives, discharged healed and deceased individuals, respectively). The representation of the Northern (blue) and Southern (red) modules from the allegiance matrix and of the swing regions (green), respectively, on the Italian map (central panel) and on the graph (right panel). Notice that we have chosen the interval [0, 1] for the Covidome because all the values are positive.

The modularity analysis on the Covidome for the hospitalized with symptoms and new positives (Fig 2A and 2B) subdivided Italy into two different modules, with a prominent North-South gradient, for both the hospitalized with symptoms and new positives indicators. The results for the remaining four time series are reported in S4 Fig of S1 File. Starting from the six different consensus matrices, obtained for each time series, we can compute the Covid “allegiance matrix” (Fig 2C, first panel). That is, the probability for two regions of being in the same community across all Covid indicators. For both within-indicator modularity, as well as for the allegiance matrix, the Covidome network is mainly split into a North-South community pattern, with some exceptions: specifically, FVG (Friuli Venezia Giulia) and Veneto are included in the “Southern” module for the hospitalized with symptoms, whereas for new positives the “Northern” community spreads over to Abruzzo and Campania, and then “Southern” incorporates Emilia Romagna and Marche in its community. Note that the allegiant community structure preserve the North-South gradient (with the exception of FVG). Interestingly, two regions had the lowest within-module allegiant score: Veneto and Umbria. That is, these regions swing between community affiliations depending on the considered Covid indicator.

Note that the division of the Italian Covidome into two different modules is robust with different values of the resolution parameter *γ* in (2) in the [0.95, 1] range, as well as when choosing different threshold values on the Covidome matrix in the percentiles between 40% and 10% (S3 Fig in S1 File).

### Dynamic Covidome via sliding window analysis

**Fig 3:**
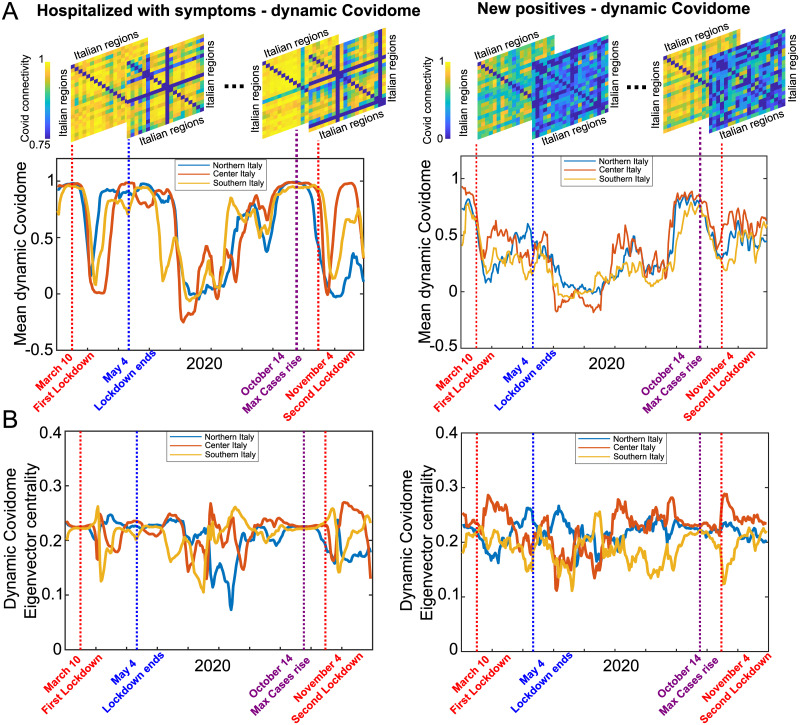
Dynamic Covidome via sliding time window analysis. **A**. First row, four different dynamic Covidomes corresponding to 10th of March, 4th of May, 14th of October and 4th of November, respectively (dashed lines). Second row, time series for the mean of the upper triangular dynamic Covidomes for three different Italian areas (first column: hospitalized individual with symptoms time series; second column: new positives; note that the dynamic Covid connectivity snapshots have different colorbar range for the two time series). **B**. Nodal Eigenvector centrality of the Dynamic Covidomes, averaged across the three Italian areas (first column: hospitalized individual with symptoms time series; second column: new positives).

The modularity analysis refers to the Covidomes computed over the pandemic period ranging from the 24th of February, 2020 to the 7th of January, 2021. In order to better investigate the associations between Covid connectivity and political decisions, we decided to perform a sliding window analysis, by computing Covidome snapshots in overlapping time windows of 21 days (see Methods for details). The results for the hospitalized individual with symptoms and the new positives Covid time series are reported in Fig 3, whereas the dynamic Covidome changes in time are represented in S1 and S2 Videos (see Supplementary material). We analyzed the dynamic Covidomes evolution for these two time series, with respect to four important dates in the pandemic policy changes: 10th of March (first national lockdown); 4th of May (restoration of freedom of movement); 14th of October (new Covid-19 positives exceeded the peak of the March infections); 4th of November (lockdown differentiated by regions). It is worth noting that a day before the third date, the 13th of October, the obligation to wear masks in open and closed public spaces was introduced. This is a key date in terms of the political decisions made (see S1 File). The mean value of the dynamic Covidome is depicted in Fig 3A, for three different Italian areas corresponding to Northern, Center and Southern Italy; Fig 3B shows the nodal eigenvector centrality of the dynamic Covidomes averaged across Northern, Central and Southern Italy.

In order to compare the two different political choices made during the first and second wave respectively, we consider the time series introduced in Fig 3 on two different time intervals. The first one considering 10 days before and 30 days after 10th of March, called *W*_1_, while the second one corresponding to 4th of November, called *W*_2_; *W*_1_ and *W*_2_ are time intervals. For both *W*_1_ and *W*_2_ we compute the minimum and maximum values for Northern, Central and Southern areas, respectively. In Table 1 we represented this values for all the mean dynamic Covidome time series introduced in Fig 3. Note that the maximum values for the new positives time series for *W*_2_ does not correspond to the absolute maximum values of the second wave, due to the fact that this values fall short before the considered range. This could be due to the wearing mask measure introduced on 13th of October, as well as the measures taken on 18th and 24th of October, before the second lockdown started. Analyzing the results related to hospitalized individuals with symptoms indicator we can see how the minimum value of the Northern time series in *W*_2_ is much lower, and negative, than the one of *W*_1_. The three minimum values in *W*_2_ correspond to 14–19 days after the second lockdown, differently form 18–30 days in *W*_1_. Another important difference between the first and second lockdown is the clear difference in between the time windows shortly after *W*_1_ and *W*_2_, respectively. In fact, for the differentiated lockdown, corresponding to the second wave, we have that the correlation between Northern regions (max = 0.3585) increase less than Southern (max = 0.7548) and Central (max = 0.9824) regions, respectively (values not reported in Table 1 but can be read from Fig 3A, the max values shortly after W2). This result cannot be seen for the first lockdown where all three the time series in the time window short after *W*_1_ reach values close to 1. Analyzing the results related to new positives time series, in the same ranges of time introduced before, we can see that only the minimum values stays in *W*_1_ and *W*_2_ respectively, moreover these values are reached from 5 to 10 days after the first lockdown while from −5 to 4 days before and after the second lockdown, respectively. From the eigenvector centrality time series Fig 3B we can see how for a short interval of time before and after the lockdown days all three Northern, Central and Southern regions remain almost constants for the hospitalized individuals with symptoms while there is a higher variability in the interval of time shortly after the first and second lockdown respectively.

**Table 1:** Maximum and minimum values for the time windows *W*_1_ = 1th March-9th April and *W*_2_ = 26th October-4th December corresponding to a range of 10 days before the first and second lockdowns and 30 days after, respectively, for mean dynamic Covidome time series (Fig 3). HS–hospitalized with symptoms, NP–new positives.

	*W*_1_ (HS)		*W*_2_ (HS)		*W*_1_ (NP)		*W*_2_ (NP)	
	min	max	min	max	min	max	min	max
Northern	0.1595	0.9551	−0.0283	0.9305	0.0789	0.8141	0.2934	0.4296
Central	0.0063	0.9795	−0.0072	0.9803	0.2834	0.8805	0.3714	0.7378
Southern	0.0811	0.9522	0.1358	0.9650	0.1596	0.7824	0.1867	0.5443

In Supplementary material are reported the results about mean dynamic Covidomes and eigenvector centrality for the remaining time series, see S5 Fig in S1 File. From Fig 3A we see that the four important dates correspond to moments of high correlation between regions, while from Fig 3B they seem to correspond to moments of temporal stability of the eigenvector centrality. We observe a similar behavior for hospitalized in ICU, home isolations, discharged healers and decesead, but we do not see the same behavior for new positives. This difference might be due to the fact that correlation values for new positives are fluctuating more with respect to the other time series considered.

### Covidome and structural connectome

**Fig 4:**
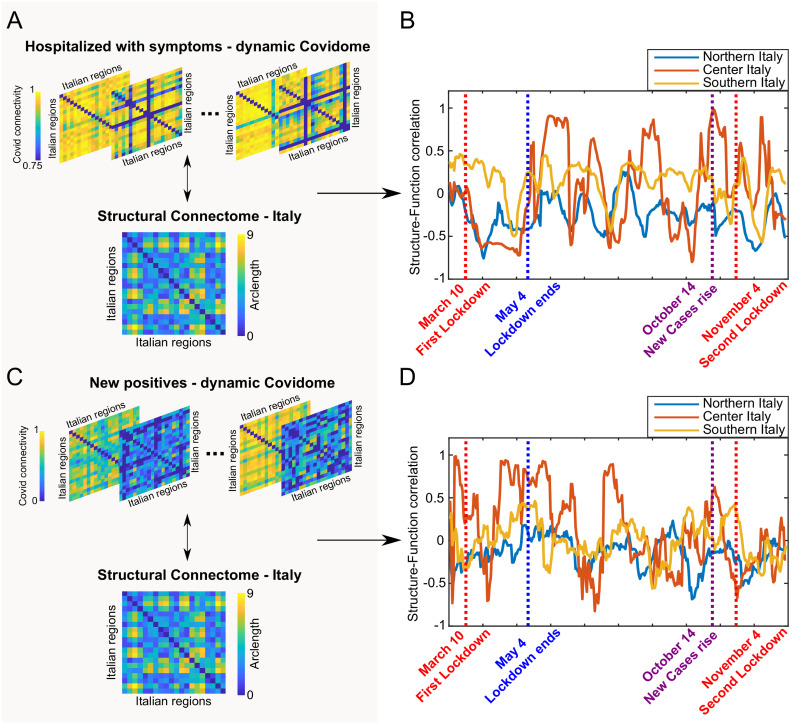
Covidome and structural connectome. **A**. Dynamic Covidomes (top row) and structural connectome for the geographical distance between Italian regions for hospitalized individual with symptoms. **B**. Time series correlation between three different sub-matrices of dynamic Covidome and structural connectome, respectively, corresponding to Northern, Central and Southern Italy for the Covid indicator introduced in A. **C**. Dynamic Covidomes (top row) and structural connectome for the geographical distance between Italian regions for new positives. **D**. Time series correlation between three different sub-matrices of dynamic Covidome and structural connectome, respectively, corresponding to Northern, Central and Southern Italy for the Covid indicator introduced in C. Notice the different range in the Covid connectivity between the tow indicators.

As our last result we analyzed the dynamic correlation between Covidome and structural connectome of Italy, in order to understand if and how Covid-19 data trend and the geographical distribution of the Italian regions were related. To this aim, we decided to correlate the dynamic Covidome snapshots (top row of Fig 4A and 4C) with the structural connectome obtained by computing the arclength of the geographical coordinates (latitude and longitude) between two different Italian regions (bottom row of Fig 4A and 4C). Fig 4B and 4D depict the temporal correlation between dynamic Covidomes and structural connectome for Northern, Center and Southern Italy areas. Please see S1 File for the results on the remaining time series (S6 Fig in S1 File).

We once more consider *W*_1_ and *W*_2_ introduced before in order to quantify the results from Fig 4B and 4D and analyze if two different lockdowns resulted in different functional-structural Covid correlation. Furthermore, there is also an evident change between the three time series fluctuation in time. As can be seen in Table 2 (for hospitalized with symptoms) the Northern regions, in both *W*_1_ and *W*_2_, are negative correlated in mean that remain negative also considering the corresponding standard deviation (std), while Central regions passes from a negative mean in *W*_1_ with lower std in absolute value to a positive one in *W*_2_, with higher std. For Southern regions there is a positive mean value in *W*_1_ and a negative one for *W*_2_. Moreover for new positives both Northern and Southern has negative mean correlation values, while Central regions, positive in the first wave and negative in the second one, respectively. The higher variability (std = 0.437) can be seen in *W*_1_ for new positives in Central regions while the lower one (std = 0.078) for Northern regions, for the same time interval.

**Table 2:** Mean and standard deviation values for the time windows *W*_1_ = 1th March-9th April and *W*_2_ = 26th October-4th December corresponding to a range of 10 days before the first and second lockdowns and 30 days after, respectively, for functional-structural correlation (Fig 4B and 4C). HS–hospitalized with symptoms, NP–new positives.

	*W*_1_ (HS)		*W*_2_ (HS)		*W*_1_ (NP)		*W*_2_ (NP)	
	mean	std	mean	std	mean	std	mean	std
Northern	−0.328	0.244	−0.380	0.182	−0.209	0.078	−0.263	0.146
Central	−0.328	0.282	0.2019	0.303	0.225	0.437	−0.257	0.205
Southern	0.317	0.094	−0.086	0.296	−0.056	0.156	−0.016	0.279

## Discussion

In this study we employed network science tools [13, 27] to study Covid-19 pandemic data one year after the Italian outbreak. Specifically, we decided to compute and study Covid connectivity (i.e., the Covidome), that is, the covariance matrix of the Covid pandemic network, across six different indicators (number of hospitalized in ICU, hospitalized with symptoms, individuals in home isolation, new positives, discharged healed and deceased). The aim was to study whether its dynamics was related to the political choices made. We have found that: (i) the Covidome community structure shows a well defined North-South pattern; (ii) dynamic Covidome fluctuations stem from the effects of the two different preventive measures during the first and the second waves, in the early and towards the end of 2020, respectively; (iii) the association between Covidome and structural constraints for mobility depends on the differences between the two different lockdowns: one nationwide, the other more localized regionally. Below follows the in-depth analysis of these findings.

### Geographical gradients of the Covidome community structure

We observed a sharp subdivision between North and South of Italy, persistent across time series and corresponding Covidomes (Fig 2A–2C and S4 Fig in S1 File). It is worth noting that this geographical pattern is obtained purely from the community structure of the Covidome, hence without considering any explicit geographical information. Nonetheless, there are few regions that do not participate to the North-South gradient: Friuli Venezia Giulia (FVG), a Northern region that gets assigned to the Southern module (Fig 2A–2C); Veneto and Umbria, respectively a Northern and a Central region, oscillate between the Norther and Southern module across Covid indicators, therefore denominated as “swing regions” (Fig 2C). The fact that FVG Covidome behavior is more related to the one of the Southern regions is not surprising. In fact, the regional administration imposed severe restrictions, such as banning public gathering, schools closure, mandatory quarantine for people from epidemiological risk areas, etc., valid already from 1st of March when the virus wasn’t circulating yet in this region [28]. Such severe regulations were adopted as well in the South of Italy. Moreover, Umbria being a swing region might be linked to two main political decisions: the first is that the first official document on the restrictions due to Covid-19 was already introduced on 26th of February, a couple of days after the Codogno case, [29]; the second, that on 4th of March more severe restrictions were introduced, since in the second wave Umbria was more affected than in the first one. Furthermore, its central position between Northern and Southern modules might also be related to its “swing region” behavior. In fact, from the Fig 2A and 2B and S4 Fig in S1 File one can notice that Umbria region is in the “Northern” module for new positives, home isolation and discharged healed individuals and in the “Southern” module for the remaining three time series. Similarly, the oscillation of Veneto across community may be due to the fact that one of the first Italian outbreaks happened in Vo’ which was, quickly, completely isolated form the rest of the region when the first cases appeared [30, 31], and on the other side, to the massive control of the people, [30–33], that might have helped in identifying people infected with Sars-Cov-2 before reaching the hospitals with more severe symptoms. In fact from Fig 2A and 2B and S4 Fig in S1 File it can be seen that Veneto region is in the “Northern” module for hospitalized in ICU and deceased individuals time series, while it falls in the “Southern” module for the remaining four indicators. We further tapped into the link between policy regulations and Covidomes, by analyzing the dynamics of the Covidomes and its relationship with the italian structural network.

### Nationwide versus region-wide lockdown impact on Covid connectivity

The Italian region mostly affected by Covid-19 was Lombardia, followed by other Northern regions, especially in the first wave of the pandemic, at the beginning of 2020. In the second wave, at the end of 2020, the pandemic spread over the entire country. The political decisions made during that year were different between the two pandemic waves, with a more severe national lockdown at the beginning of 2020, followed by a region-specific lockdown for the second wave. These two different political approaches propagate into the Italian Covidome dynamics. Fig 3 shows that the average minimum values for dynamic Covidomes of the hospitalized individuals with symptoms appear at distance of 18, 30 and 23 days after 10th of March (first lockdown) for Northern, Central and Southern areas. In contrast, during the second lockdown the decrease happens at 17, 19 and 14 days after the 4th of November. Interestingly, the mean value of dynamic Covidome increases after the second lockdown, and it is higher for the Central Italian regions, followed by Southern and Northern Italy, respectively. It is evident from these findings that a lockdown leads to low Covidome values between regions across all Covid indicators, with the exception of discharged healed and deceased individuals: this might be due to the fact that these time series represent the cumulative numbers in time, hence there is no first and second wave trend as the time series are only increasing. In fact we did the simulations by using daily values and we noticed that for both discharged healed and deceased individuals time series we have more variation in the mean dynamic Covidome values than for the case where cumulative numbers were used, with the exception of the last one that leads in any case to high values of the mean dynamic Covidome. Furthermore, the difference between first and second wave may be due to the fact that during the first lockdown most of the cases were concentrated in the Northern regions and hence the situation was more heterogeneous, while in the second wave different regions were experiencing similar lockdown scenarios (values of Covidome are indeed not as low as in the first wave). For all six time series we observed another correlation drop during summer, when there were less Covid cases, and in all the cases but home isolation the lower values were localized in this time interval. Notably, the drop in the dynamic Covidome values soon after the lockdowns appears with a certain delay with respect to the effective dates of the hospitalized individuals with symptoms, and no delay for new cases. The reason for this different behavior is two-fold: first, the information at each time point in Fig 3 represents a window of three weeks; second, it was shown that there is an intrinsic delay of 3 and 10.4 days, depending on the age of the patient, from the day a person shows symptoms to Sars-Cov-2 and needs to be hospitalized [34].

Note that the dynamic Covidome fluctuations should always be interpreted together with the historical process of the pandemic spread. For instance, we observed three characteristic drops in the average Covidome value in most of the time series considered, specifically hospitalized with symptoms, new positives, hospitalized in ICU and home isolation: two, shortly after the lockdowns, when the virus slowed down due to the prevention measurements taken; the third one, during the summer, when no restriction traveling was imposed. The summer drop might have a trivial explanation, intrinsic to the pandemic evolution in time: it can be related to a trend towards zero of the aforementioned indicators, which will consequently bring the average Covidome value to zero as well. This is probably due to the fact that people gathered outside, diminishing the probability of getting infected. Recent studies also found a negative correlation between the external temperature and the spreading of the virus [35, 36]. The two drops after the lockdown measures were taken, however, leave room for more interesting speculations. These decreases suggest that the covariance between pairs of Italian regions break off. That is, the regional indicators’ trends get decoupled with each other, after the two lockdown measures were introduced (see also S1 and S2 Videos of Supplementary material). Hence, we can conclude that the decrease in the average Covidome value after the first and second wave (see S2A1-S2F1 Fig in S1 File) might be a consequence of how each region has dealt with the two policy measurements, which in turn has brought the synchrony in the pandemic indicators to fade. Hence, two similar drops might have different meanings, depending on the political choices adopted and the level of diffusion of the pandemic.

### Dynamic Covidome and structural connectome correlation

From the functional-structural correlation results (Fig 4B) one can notice that the Covid connectivity of the Northern regions is generally poorly correlated to the structure, whereas the Central regions change from high to low correlation (≃[−1, 1]) during the pandemic outbreak, and finally the Southern regions vary their structure-function correlation in smaller interval than the Central regions (≃[−0.5, 0.5]). During the first wave the dynamic Covidomes of the hospitalized with symptoms (Fig 4B) of the Southern regions are positively correlated with the structural connectome. Northern and Central regions, however, both show little or no correlation with the geographical Italian network during the first wave. The scenario changes completely when one analyzes the hospitalized with symptoms during the second wave. The Covid connectivity of the Central regions co-varies with interregional distance in a larger range, as opposed to the Northern regions where the functional-structural correlation is always negative.

For what concerns the structure-function associations between dynamic Covidomes of new positive cases (Fig 4D), it is noticeable a larger variability (≃[−1, 1]) for the Central regions, whereas this range gets smaller for Northern and Southern areas. The variability for central regions decrease in the second differentiated lockdown. These results also confirm the score of the Central regions in the Covid allegiance matrix. As can be seen in Table 2, for new positives, in the first wave there is a negative functional-structural correlation average for the Northern and Southern regions, respectively, which is maintained across the second wave.

Two hypothesis can be postulated to explain the structure-function differences across lockdowns: one relates to the fact that the virus outbreak originated in the North and hit the Southern region with a larger delay, due to the closure of the borders; the second is that, during the second wave, the political decisions aimed at reducing the mobility from and to the “at risk” regions helped in “disconnecting” the Covidome dynamics from the Italian geographical network, hence keeping the functional-structural correlation mean negative.

### Limitations and future directions

This study has some limitations. The use of the Pearson’s correlation coefficient as a metric to compute the Covidomes might be limited. Further studies should explore more advanced methods or directed measurements, based on information theory and time series analysis, or even, for a deeper analysis, on Graph Signal Processing tools [37, 38]. Another limitation, that can also bring to further analysis, is the computation of the structural connectome based on the geographical arclength between regions. It will be interesting to see how the Covidome relates to the structural connectome based on mobility data [39], or even the one extracted from the Italian public transportation network data.

Furthermore, in our work, the community detection was performed over the entire 2020 time-series. Assuming that regions behaved similarly during the first lockdown and differently during the second, due to the different lockdown policies, it could be interesting to separate the two periods of analyses. Future studies might use the first lockdown as a “null model” (no regional differentiation on the containment policy), and use it as a benchmark for the analysis of second lockdown (where effective differentiation happens).

Here we have used the eigenvector centrality of a correlation matrix in order to test the functional “prominence” or relevance of a specific region/area in the Covid functional connectivity, and how that is related to the political policies. Future work should also explore other centrality measures.

The Covidome methodology presented here and applied to the Italian case study can be easily adapted to other countries with a federal state organization, or more generally at the European level, depending on the granularity detail of study. The analysis on the second lockdown could also be performed using data at Italian provincial level, similarly to [40]. Besides the aforementioned limitations, we believe that the use of these network-scientific tools might inform on the link between the temporal information inherent to Covid time series and the efficiency of the political decisions of each nation’s governments.

### Conclusions

We here presented a first investigation of the functional network of Covid-19 pandemic (Covidome), across different indicators. We show that dynamics and structure of the Covidomes is dependent of the political choice made by the Italian government, suggesting that the Covidome might serve as a good indicator to infer region-to-region spreading during the pandemic. This approach seems promising based on these preliminary findings, and we hope that it can help in shedding light on the complex system generated by Covid-19.

## Supporting information

S1 File Supplementary material. In this file we report the results for the remaining four time series, hospitalized in ICU, home isolation, discharged healed and deceased individuals, respectively. Moreover the robustness of the modularity solutions on different parameters is introduced.
(ZIP)

S1 Video Hospitalized with symptoms dynamic Covidome. On the top row we have represented the dynamic Covidome (left panel) and the Italian regions map (right panel) containing the normalized (to [0, 1] interval) regional average connectivity of the dynamic Covidome in time. On the bottom row we have plotted the three time series corresponding to the mean dynamic Covidomes for the aforementioned Italian areas. The green sliding window depicts the 21 days time window.
(AVI)

S2 Video New positives dynamic Covidome. On the top row we have represented the dynamic Covidome (left panel) and the Italian regions map (right panel) containing the normalized (to [0, 1] interval) regional average connectivity of the dynamic Covidome in time. On the bottom row we have plotted the three time series corresponding to the mean dynamic Covidomes for the aforementioned Italian areas. The green sliding window depicts the 21 days time window.
(AVI)
