# Supplementary material for: How political choices shaped Covid connectivity: The Italian case study
Source: PLoS One. 2021 Dec 10;16(12):e0261041. doi: 10.1371/journal.pone.0261041 (PMC8664190; doi:10.1371/journal.pone.0261041)
Supplement: S1 File — In this file we report the results for the remaining four time series, hospitalized in ICU, home isolation, discharged healed and deceased individuals, respectively. Moreover the robustness of the modularity solutions on different parameters is introduced. (ZIP) [file pone.0261041.s001.zip › Supplementary.pdf]

Supplementary material

Legend Figure 1: **Workflow of the Covid connectivity analysis for hospitalized individuals with symptoms in Italy, during 2020. A.** The time series of hospitalized individuals with symptoms for all the 20 Italian regions. **B.** The *Covidome* (the adjacency matrix of the network) obtained by computing the Pearson's correlation coefficients associated to data reported in panel A. **C.** Modularity of the Covidome for the considered time series, represented both on the Italian map (left panel) and on the graph (right panel), respectively. **D.** Average Covid connectivity obtained using sliding window correlation. The three different curves represent three different areas corresponding to Northern, Central and Southern Italy.

Legend Figure 2: **Community structure of the Italian Covidome. A.** The Covidome partition, after consensus clustering, for the hospitalized with symptoms time series, represented on the map (left panel) and on the Covidome graph (right panel). **B.** The Covidome partition, after consensus clustering, for the new positives time series, on the map (left panel) and on the Covidome graph (right panel). **C.** The Covidome allegiance matrix (left panel) across the six different Covid indicators (i.e., number of hospitalized individuals in ICU, number of hospitalized individuals with symptoms, number of individuals in home isolation, new positives, discharged healed and deceased individuals, respectively). The representation of the Northern (blue) and Southern (red) modules from the allegiance matrix and of the swing regions (green), respectively, on the Italian map (central panel) and on the graph (right panel).

Legend Figure 3: **Dynamic Covidome via sliding time window analysis. A.** First row, four different dynamic Covidomes corresponding to 10th of March, 4th of May, 14th of October and 4th of November, respectively (dashed lines). Second row, time series for the mean of the upper triangular dynamic Covidomes for three different Italian areas (first column: hospitalized individual with symptoms time series; second column: new positives; note that the dynamic Covid connectivity snapshots have different colorbar range for the two

time series). **B.** Nodal Eigenvector centrality of the Dynamic Covidomes, averaged across the three Italian areas (first column: hospitalized individual with symptoms time series; second column: new positives).

Legend Figure 4: **Covidome and structural connectome. A.** Dynamic Covidomes (top row) and structural connectome for the geographical distance between Italian regions for hospitalized individual with symptoms. **B.** Time series correlation between three different sub-matrices of dynamic Covidome and structural connectome, respectively, corresponding to Northern, Central and Southern Italy for the Covid indicator introduced in A. **C.** Dynamic Covidomes (top row) and structural connectome for the geographical distance between Italian regions for new positives. **D.** Time series correlation between three different sub-matrices of dynamic Covidome and structural connectome, respectively, corresponding to Northern, Central and Southern Italy for the Covid indicator introduced in C. Notice the different range in the Covid connectivity between the tow indicators.

Legend Table 1: Maximum and minimum values for the time windows $W_1 =$ 1th March-9th April and $W_2 =$ 26th October-4th December corresponding to a range of 10 days before the first and second lockdowns and 30 days after, respectively, for mean dynamic Covidome time series (Fig.3). HS–hospitalized with symptoms, NP–new positives.

Legend Table 2: Mean and standard deviation values for the time windows $W_1 =$ 1th March-9th April and $W_2 =$ 26th October-4th December corresponding to a range of 10 days before the first and second lockdowns and 30 days after, respectively, for functional-structural correlation (Fig.4-B and C). HS–hospitalized with symptoms, NP–new positives.

Fig.S1 shows the geographical subdivision in Northern (blue), Central (green) and Southern (red) of the Italian regions, that corresponds to the macro-areas studied in the main text for the time series shown in Fig. 3-4.

Fig.S2 depicts all six different time series considered for our analysis: the number of hospitalized individuals in intensive care units (ICU), hospitalized individuals with symptoms, individuals in home isolation, new positives, dis-

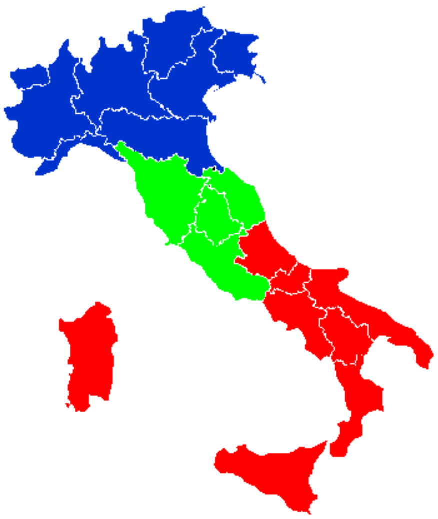

Figure S1: **Three Italian areas.** Geographical subdivision in Northern (blue), Central (green) and Southern (red) of the Italian regions.

charged healed and deceased individuals, as well as the corresponding Covidomes, which are described in the main draft.

In the main draft we studied the modularity of the Italian Covidome network (see Materials and Methods for details). In Fig.S3 we tested the robustness of the modularity solutions on different parameters. Panel A of Fig.S3 represents the percolation density of Covid networks, that is, the proportion of nodes in largest components in function of different thresholds (i.e., connection density). Note how the Covidomes appear to be quite stable, unless a very aggressive threshold is chosen (below 50%). Furthermore, in order to test the dependency of the reported modularity analysis on the resolution parameter γ, we reported in panel B and C the changes in the number of modules and modularity score Q_{score} for different values of γ. Note that $\gamma = 1$ (which is usual the "default" value for the Louvain algorithm) seems to be a reasonable choice, since for all the Covid indicators the Q_{score} is consistently high, and up to this value of γ we get a stable partition of two main modules (after that, it breaks down into individual modules).

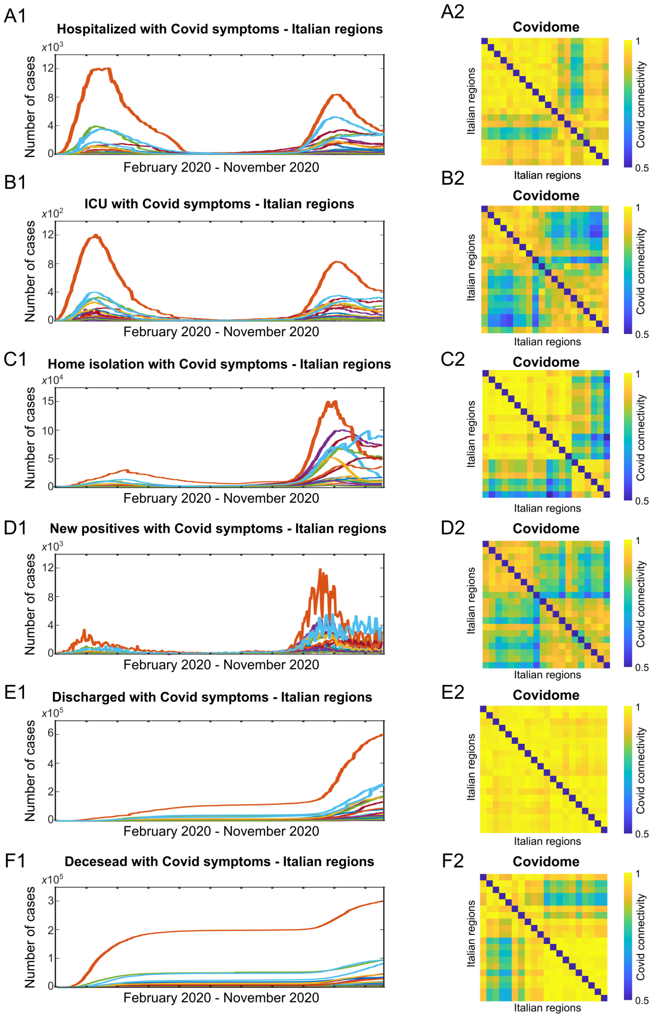

Figure S2: **Six time series and the corresponding Covidomes. A1.** The time series of hospitalized individuals with symptoms for all 20 Italian regions. A2. The Covidome (the adjacency matrix of the network) obtained by computing the Pearson's correlation coefficients associated to data reported in panel A1. **B1.** The time series of hospitalized individuals with symptoms in ICU for all 20 Italian regions. B2. The Covidome associated to data reported in panel B1. **C1.** The time series of home isolation individuals with symptoms for all 20 Italian regions. C2. The Covidome associated to data reported in panel C1. **D1.** The time series of new positives for all 20 Italian regions. D2. The Covidome associated to data reported in panel D1. **E1.** The time series of discharged individuals for all 20 Italian regions. E2. The Covidome associated to data reported in panel E1. **F1.** The time series of deceased individuals for all 20 Italian regions. F2. The Covidome associated to data reported in panel F1.

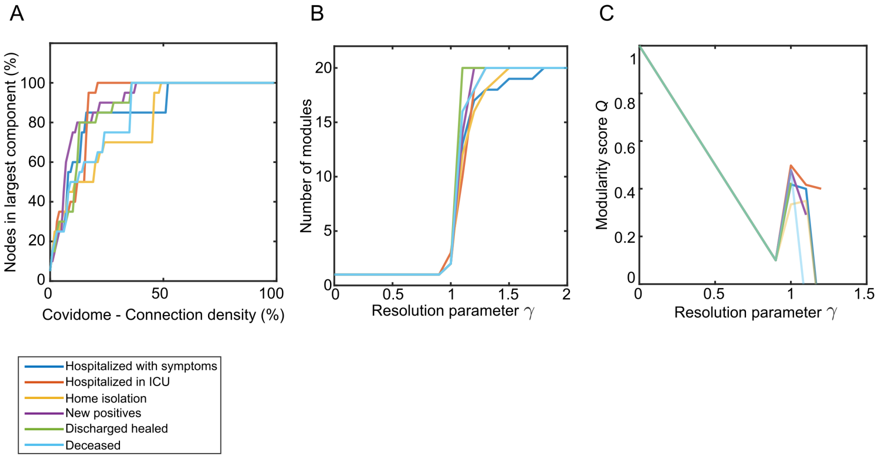

Figure S3: **Robustness of the Covidome modularity analysis.** **A.** Connection density versus nodes in largest components for all six Covid indicators series. **B.** The dependency of the number of modules on different choices of γ for all six time series. **C.** The dependency of the Q_{score} on different choices of γ.

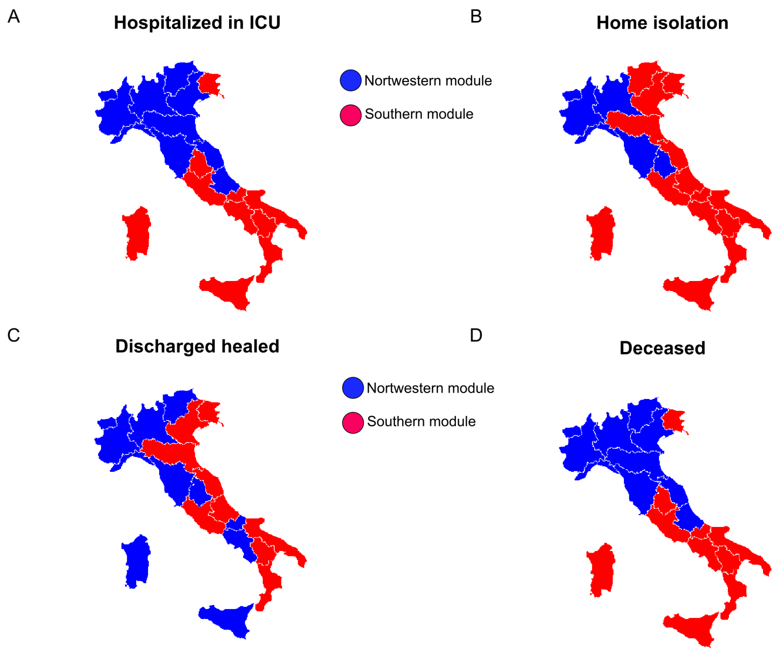

Figure S4: **Consensus modularity A.** The consensus partition for the hospitalized in ICU time series on the map. **B.** The consensus partition for the home isolation individuals time series on the map. **C.** The consensus partition for the discharged healed individuals time series on the map. **D.** The consensus partition for the deceased individuals time series on the map.

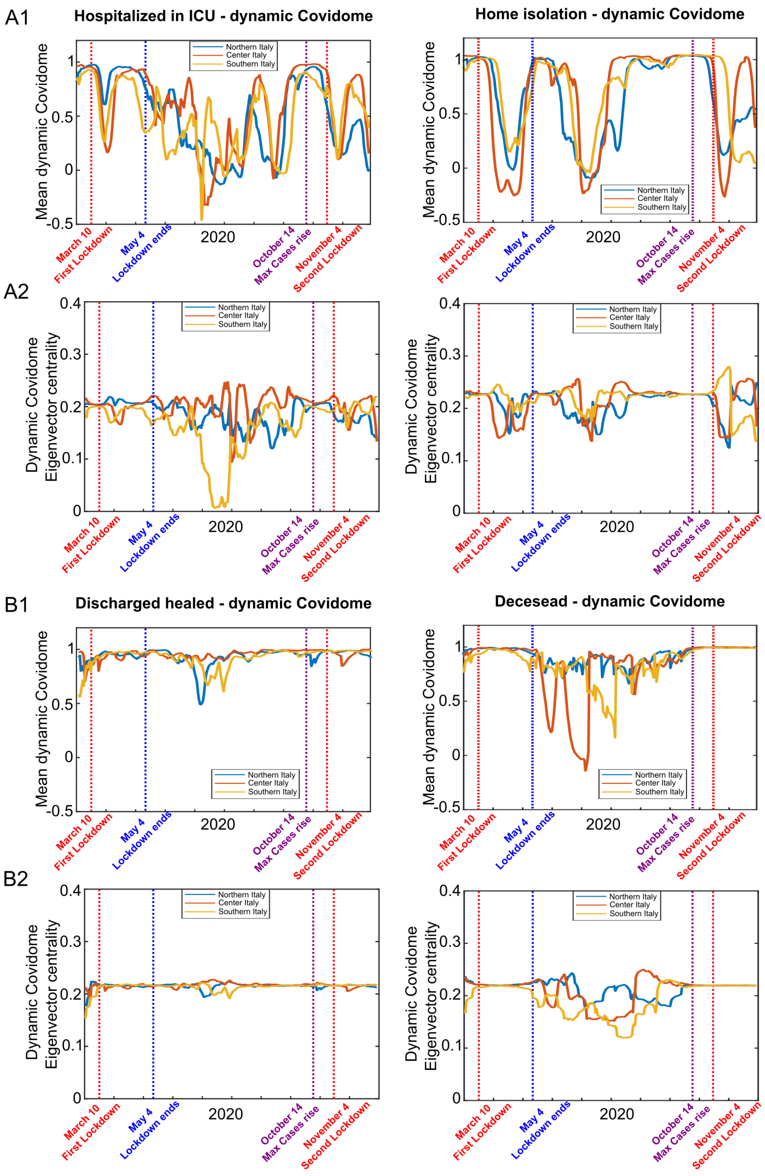

Figure S5: **Dynamic Covidome via sliding time window analysis. A1, B1.** Time series for the mean of the upper triangular dynamic Covidomes for the three different Italian areas (depicted in Fig. S1). **A2, B2.** Eigenvector centrality of the dynamic Covidomes for the three Italian areas. In **A1** and **A2** the first column refers to hospitalized in ICU and the second column to home isolation, whereas in **B1** and **B2** the first column refers to discharged healed and the second column to deceased individuals.

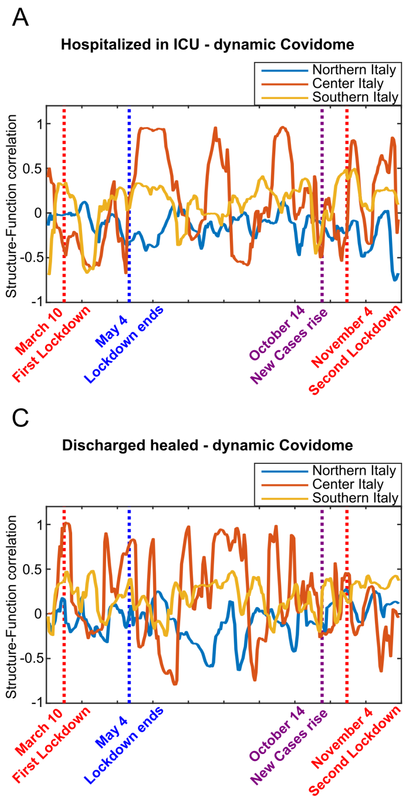

Figure S6: **Covidome and structural connectome correlation.** Correlation between dynamic Covidomes and structural connectome for Northern, Central and Southern Italy, relative to the **A)** hospitalized in ICU time series. **B)** the home isolation individuals time series, **C)** the discharged individuals time series, and **D)** the deceased individuals time series.

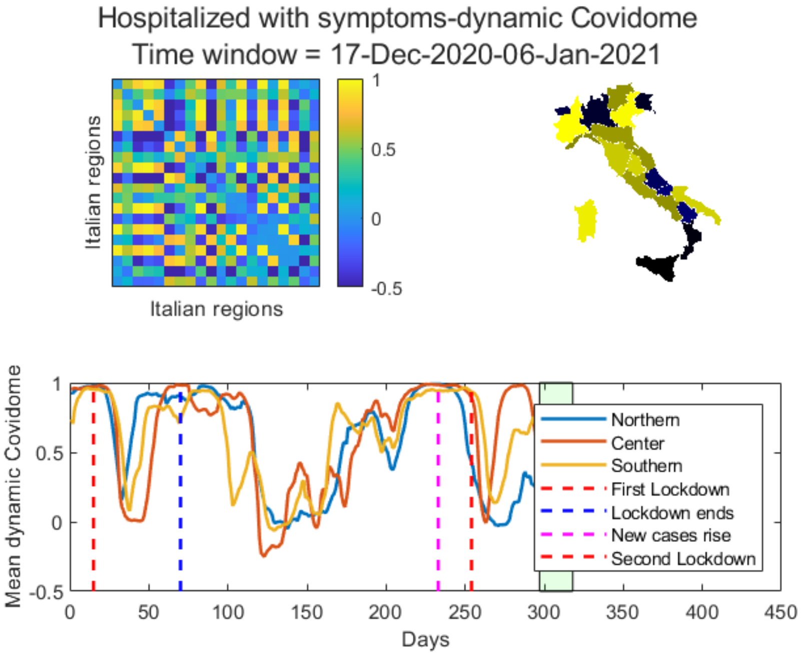

Figure S7: **Snapshot of Video 1**. Explanatory snapshot of Video 1 (same for Video 2 but for a different time series). On the top row we have represented the dynamic Covidome (left panel) and the Italian regions map (right panel) containing the normalized (to $[0,1]$ interval) regional average connectivity of the dynamic Covidome in time. On the bottom row we have plotted the three time series corresponding to the mean dynamic Covidomes for the aforementioned Italian areas. The green sliding window depicts the 21 days time window.
